# Regional Conference on Emerging Infectious Diseases Sparks Plan for Increased Collaboration

**DOI:** 10.3201/eid0201.960120

**Published:** 1996

**Authors:** Bradford A. Kay


### CDC Convenes Meeting to Discuss Strategies for Preventing Invasive Group A Streptococcal Infections

Since the mid-1980s, the epidemiology of invasive group A streptococcal (GAS) infections in the United States and worldwide has changed, and the incidence of invasive infections, streptococcal toxic shock syndrome (strep TSS), and necrotizing fasciitis has increased. These changes may be the result of a shift in GAS M-types and a corresponding increase in strains that produce certain pyrogenic exotoxins. Recognizing the importance of monitoring changes in the occurrence of severe group A streptococcal disease, the Council of State and Territorial Epidemiologists recommended in April 1995 that invasive GAS infections and strep TSS be added to the National Public Health Surveillance System.

Most invasive GAS infections occur sporadically and are acquired in the community. For these cases, preventing illness and death depends on improving recognition and treatment. Primary prevention of invasive GAS disease may be more feasible for infections that are acquired in institutions (such as hospitals and nursing homes) and for secondary cases that occur among contacts of persons with invasive disease. Most nosocomial infections (for example, wound infections, postpartum endometritis, and sepsis) occur in surgical or obstetric settings, or are associated with intravenous catheters. Secondary invasive disease in the community is uncommon, although studies of household contacts of those with GAS infectionshave found a substantially increased risk for infection in this group. GAS infections spread easily from person to person after contact with respiratory secretions of an infected person and have traditionally caused epidemics of pharyngitis, scarlet fever, and rheumatic fever. Recently, clusters of invasive infections have been reported in families, hospitals, and nursing homes; community-wide outbreaks have also been reported.

As state health departments initiate surveillance for invasive GAS disease and strep TSS, guidelines for prevention will help in interpreting these data and in formulating a public health response. CDC convened a meeting of experts from academia and public health (October 10-11, 1995), to discuss existing data and strategies for preventing invasive GAS disease in institutions and the community. Discussions centered on the magnitude of risk for secondary disease among close contacts of persons with invasive infection and the potential for preventing disease by chemoprophylaxis, and on approaches for investigating and preventing infections in institutions. Recommendations are being developed, and the conclusions of the participants will be presented at a later date.

**The Working Group on Prevention of Severe Group A Streptococcal Infections***
National Center for Infectious Diseases
Centers for Disease Control and Prevention
Atlanta, Georgia, USA

*The members of the Working Group on Prevention of Severe Group A Streptococcal Infections are Gus Birkhead, New York State Department of Health; John Brundage, U.S. Army; Matt Cartter, Connecticut State Department of Health; Mike Gerber, University of Connecticut; Walter Hierholzer, Yale University; Ed Kaplan, University of Minnesota; Kris MacDonald, Minnesota Department of Health; Dennis Stevens, V.A. Medical Center, Boise, Idaho; Karen Green and Allison McGeer, Princess Margaret Hospital, Toronto, Ontario, Canada; Stan Shulman and Ram Yogev, Children's Memorial Hospital, Chicago, Illinois; Richard Facklam, Julia Garner, William Jarvis, Orin Levine, Benjamin Schwartz and Jay Wenger, CDC.

### Regional Conference on Emerging Infectious Diseases Sparks Plan for Increased Collaboration

The World Health Organization (WHO), the Naval Medical Research Unit Three (NAMRU-3), and the Centers for Disease Control and Prevention (CDC) jointly sponsored the first conference in the region on issues of emerging and reemerging infectious diseases for members of WHO's Eastern Mediterranean Regional Office (EMRO). The meeting was held in Cairo, Egypt, November 26-29, 1995. Delegates from the WHO South-East Asia Regional Office and African Regional Office also participated.

The meeting brought together persons representing key resources that have begun working together to organize a regional program of laboratory assistance and enhanced surveillance communications for infectious diseases. Participants included WHO infectious disease program officers and key personnel from WHO collaborating centers, national reference laboratories, national infectious disease programs, ministries of health, and university public health programs.

The World Health Organization (WHO), the Naval Medical Research Unit Three (NAMRU-3), and the Centers for Disease Control and Prevention (CDC) jointly sponsored the first conference in the region on issues of emerging and reemerging infectious diseases for members of WHO's Eastern Mediterranean Regional Office (EMRO). The meeting was held in Cairo, Egypt, November 26-29, 1995. Delegates from the WHO South-East Asia Regional Office and African Regional Office also participated.

The meeting brought together persons representing key resources that have begun working together to organize a regional program of laboratory assistance and enhanced surveillance communications for infectious diseases. Participants included WHO infectious disease program officers and key personnel from WHO collaborating centers, national reference laboratories, national infectious disease programs, ministries of health, and university public health programs. Thirty countries were represented by more than 200 participants. On the final day of the conference, the participants adopted a regional plan of action for strengthening surveillance, laboratory capabilities, and communications.

To begin this cooperative effort, a 1-day meeting of WHO-EMRO collaborating centers was convened on November 30. Representatives from six EMRO collaborating centers attended as well as representatives from WHO-EMRO, WHO headquarters, CDC, the Naval Medical Research Institute in Washington, D.C., and the Battelle Foundation.

Participants noted that WHO collaborating centers are an excellent resource for the implementation of programs to address emerging infections because they represent some of the most competent and experienced diagnostic and reference capacities in the region. However, the representatives agreed that the relationships among the collaborating centers themselves are not well developed. Many were unaware of the existence or capabilities of most other regional collaborating centers and most had no regular communications or interactions with other collaborating centers. All welcomed closer ties with an increase in communication, collaboration, and interaction.

The participants unanimously approved a recommendation to establish an Association of EMRO collaborating centers composed of representatives from each WHO collaborating center within the region. Member organizations of the association will work together, in coordination with EMRO, to address the complex needs of surveillance, laboratory diagnostics, and disease reporting of emerging infections at the local and regional level.

